# Genetic Landscape of Prostate Cancer Conspicuity on Multiparametric Magnetic Resonance Imaging: A Systematic Review and Bioinformatic Analysis

**DOI:** 10.1016/j.euros.2020.06.006

**Published:** 2020-07

**Authors:** Joseph M. Norris, Benjamin S. Simpson, Marina A. Parry, Clare Allen, Rhys Ball, Alex Freeman, Daniel Kelly, Hyung L. Kim, Alex Kirkham, Sungyong You, Veeru Kasivisvanathan, Hayley C. Whitaker, Mark Emberton

**Affiliations:** a UCL Division of Surgery & Interventional Science, University College London, London, UK; b London Deanery of Urology, London, UK; c Department of Urology, University College London Hospitals NHS Foundation Trust, London, UK; d UCL Cancer Institute, University College London, London, UK; e Department of Radiology, University College London Hospitals NHS Foundation Trust, London, UK; f Department of Pathology, University College London Hospitals NHS Foundation Trust, London, UK; g School of Healthcare Sciences, Cardiff University, Wales, UK; h Department of Urology, Cedars-Sinai Medical Center, West Hollywood, CA, USA; i Department of Biomedical Sciences, Cedars-Sinai Medical Center, West Hollywood, CA, USA

**Keywords:** Bioinformatic analysis, Genetics, Genomics, Multiparametric magnetic resonance imaging, Prostate cancer, Systematic review

## Abstract

**Context:**

Multiparametric magnetic resonance imaging (mpMRI) detects most, but not all, clinically significant prostate cancer. The genetic basis of prostate cancer visibility and invisibility on mpMRI remains uncertain.

**Objective:**

To systematically review the literature on differential gene expression between mpMRI-visible and mpMRI-invisible prostate cancer, and to use bioinformatic analysis to identify enriched processes or cellular components in genes validated in more than one study.

**Evidence acquisition:**

We performed a systematic literature search of the Medline, EMBASE, PubMed, and Cochrane databases up to January 2020 in accordance with the Preferred Reporting Items for Systematic Reviews and Meta-analyses (PRISMA) statement. The primary endpoint was differential genetic features between mpMRI-visible and mpMRI-invisible tumours. Secondary endpoints were explanatory links between gene function and mpMRI conspicuity, and the prognostic value of differential gene enrichment.

**Evidence synthesis:**

We retrieved 445 articles, of which 32 met the criteria for inclusion. Thematic synthesis from the included studies showed that mpMRI-visible cancer tended towards enrichment of molecular features associated with increased disease aggressivity, including phosphatase and tensin homologue (*PTEN*) loss and higher genomic classifier scores, such as Oncotype and Decipher. Three of the included studies had accompanying publicly available data suitable for further bioinformatic analysis. An over-representation analysis of these datasets revealed increased expression of genes associated with extracellular matrix components in mpMRI-visible tumours.

**Conclusions:**

Prostate cancer that is visible on mpMRI is generally enriched with molecular features of tumour development and aggressivity, including activation of proliferative signalling, DNA damage, and inflammatory processes. Additionally, there appears to be concordant cellular components and biological processes associated with mpMRI conspicuity, as highlighted by bioinformatic analysis of large genetic datasets.

**Patient summary:**

Prostate cancer that is detected by magnetic resonance imaging (MRI) tends to have genetic features that are associated with more aggressive disease. This suggests that MRI can be used to assess the likelihood of aggressive prostate cancer, based on tumour visibility.

## Take Home Message

The genomic evidence surrounding multiparametric magnetic resonance imaging (mpMRI) visibility is varied and often discordant; however, the literature suggests that tumour mpMRI visibility correlates with aggressive genomic features.

## 1 Introduction

Multiparametric magnetic resonance imaging (mpMRI) has enhanced risk stratification for men at a risk of prostate cancer, through accurate prebiopsy detection of clinically significant disease [1]. However, approximately 10–20% of clinically significant prostate cancers are not detected by mpMRI [1], and the nature of mpMRI-invisible disease remains a potential source of concern.

The biology underlying mpMRI conspicuity of prostate cancer is poorly understood; however, tumour visibility on mpMRI appears to be associated with disease significance and aggressivity [2]. Disease aggressivity in prostate cancer can be defined clinically in several ways, including reduced time to recurrence following treatment, time to metastasis, and prostate cancer–specific mortality. Pathologically, the Gleason grading system appears to correlate with clinical outcome, with higher-grade disease exhibiting increased features of disease aggressivity [3]. Furthermore, aggressive cancer is known to harbour particular genomic hallmarks, including *MYC* amplification, *ATM* mutation, hypermethylation of *TCERG1L* (5′ upstream), and loss of *PTEN* [4]. The potential mechanistic association of these molecular features with mpMRI phenotypes and their prognostic significance has been an area of recent research focus [5], now warranting collation.

Here, we systematically review the evidence surrounding the genomic characteristics underlying the mpMRI conspicuity of prostate cancer, for the first time. We also identify genes associated with mpMRI conspicuity, which are experimentally validated, and identify enriched pathways and functions using publicly available mpMRI-correlated genetic databases.

## 2 Evidence acquisition

### 2.1 Study design

The protocol for this systematic review and bioinformatic analysis has been published in detail elsewhere [6], and was based on the Preferred Reporting Items for Systematic Review and Meta-analysis Protocols (PRISMA-P) statement. This review was also prospectively registered in the PROSPERO International Registry (CRD42019147423).

### 2.2 Literature search

A systematic search of the literature was conducted from 1990 to 2020 in four databases: MEDLINE, PubMed, EMBASE, and Cochrane. Controlled vocabulary was selected in the search engines to reduce the number of unrelated studies. The search strategy contained 11 components linked by the AND/OR operator terms: (Prostate AND cancer) AND (gene OR genetic OR genome OR genomic OR transcriptome OR transcriptomic OR epigenetic) AND (magnetic resonance imaging OR MRI).

### 2.3 Study selection

**Fig. 1:**
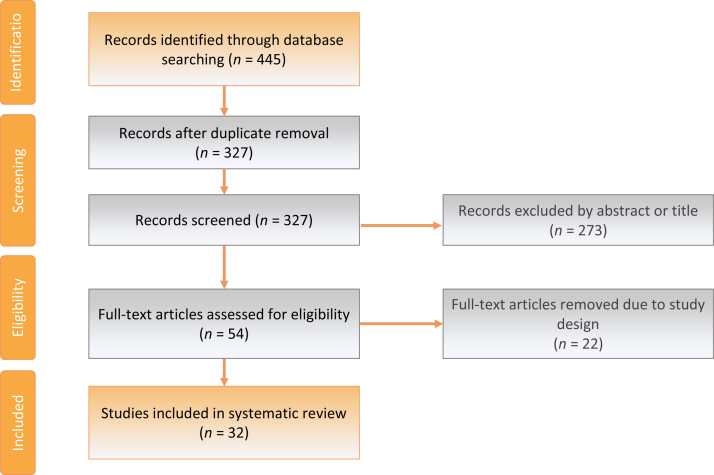
PRISMA flow diagram of evidence acquisition. PRISMA = Preferred Reporting Items for Systematic Reviews and Meta-analyses.

Figure 1 shows an overview of the evidence acquisition process. Two investigators (B.S.S. and J.M.N.) independently screened eligible studies, assessing titles and abstracts for relevance. Full texts were retrieved and reviewed further for eligibility. Lack of concordance between reviewers was discussed until consensus was reached or passed to a third reviewer (M.A.P.). For inclusion in the analysis, studies had to demonstrate investigation of the genomic aspects of localised prostate cancer conspicuity on mpMRI. Genomic investigation was at the DNA level, including larger-scale alterations (copy-number changes or methylation). Transcriptomic data analysing RNA expression (coding or noncoding) or microRNA were also included. All proteomic methodologies were accepted, including immunohistochemistry (IHC). Conference abstracts, correspondence articles, expert opinions, and case reports were excluded. Studies that did not correlate tumour visibility on mpMRI with genomic data were excluded. Articles focusing solely on clinical or histopathological features of mpMRI conspicuity were removed. Studies that focused on advanced or metastatic prostate cancer were excluded.

### 2.4 Data collection

Identified articles were uploaded to Rayyan (a web and mobile application for systematic reviews) to expedite initial screening and allow reviewers to filter studies. Reference sections of included articles were searched manually to identify missed studies and additional data. All extracted data were collected by two investigators (B.S.S. and J.M.N.) using a standardised form. Both reviewers extracted data independently and agreed by consensus.

### 2.5 Quality assessment

Risk of bias assessment was conducted using a modified Newcastle-Ottawa scale. Studies were assessed on grounds of patient selection, comparability, and outcome. Large biopsy cohorts were considered most representative, encompassing an accurate cross section of disease upon diagnosis, followed by smaller biopsy cohorts (<50 patients) and then radical prostatectomy cohorts, and finally nonrepresentative sampling from another route. The Newcastle-Ottawa scale is intended for traditional clinical outcome–focused meta-analyses, so we simplified outcome measures to a single parameter whereby the quality of genetic outcome was assessed. Unbiased whole genome, methylome, transcriptome, or proteome-based approaches were considered gold standard (two stars), followed by large-scale but limited methods based on arrays or very large gene panels (one star) and then selected gene panels (such as those used in commercial assays), and lastly, approaches that investigated single genes. The Newcastle-Ottawa scale allowed for a maximum of eight stars: four for selection, two for comparability, and two for outcome.

### 2.6 Data synthesis

The primary endpoint was differential gene expression between mpMRI-visible and mpMRI-invisible tumours. Secondary endpoints were explanatory links between gene function and mpMRI conspicuity, and potential prognostic value of differential gene enrichment. Key themes were derived from the included the literature with a focus on mpMRI scoring systems used (eg, Prostate Imaging Reporting and Data System [PI-RADS], Likert, and radiogenomic features), criteria used to define tumour visibility (usually a PI-RADS or Likert score cut-off), and the type of cohort used in the study (eg, radical prostatectomy or biopsy cohort).

### 2.7 Bioinformatic analysis

In the identified articles, there were an insufficient number of studies with single endpoints and comparable methodologies to conduct a typical meta-analysis. Therefore, an additional search was conducted to identify available genetic datasets for bioinformatic analysis in the NCBI GEO and European genome-phenome archives. For retrieved transcriptomic data, Log2-fold changes and associated false discovery rate (FDR)-adjusted values were compared between mpMRI-visible and mpMRI-invisible tumours. Differential gene expression was compared between studies; if unavailable, highlighted genomic features and the direction of change (eg, correlation coefficient) were compared between groups. Genes highlighted in multiple studies were used (via over-representation analysis) to identify enriched pathways, components, and functions. Analyses were performed using the WebGestalt, a gene set analysis toolkit. This method enables a standardised and robust analysis, as it does not rely on significance or effect size weighting (measures of effect size differed between studies) and uses a modified Fisher's exact test to identify enriched biological processes.

## 3 Evidence synthesis

### 3.1 Study characteristics

We retrieved 445 articles: 262 from EMBASE, 129 from Medline, 42 from Cochrane, eight from PubMed, and four from reference searching or expert suggestion. Of these, 32 articles were eligible for further analysis (Table 1). Of the 32 studies, 14 used prostate biopsy as the source of prostate tissue for genetic analysis, 16 used radical prostatectomy specimens, and two used a combination of these two approaches. Median study size was 51 (range 2–532). The PI-RADS reporting scheme was the most commonly used mpMRI reporting approach, with 21 of the included studies using this system or a modified version. Of those using PI-RADS, 14 used PI-RADSv2. Assessment by an expert radiologist was the second most common mpMRI scoring approach, employed in six studies, followed by scores based on radiomic-derived features, used in three of the studies. Two studies used a modified or different reporting measure. For the purpose of comparison, 12 studies chose to discretise scoring systems into “mpMRI-visible” and “mpMRI-invisible” tumours, with the exception of two studies that included an “indeterminate” category. Definitions of tumour conspicuity on mpMRI were heterogeneous between studies, with one study defining visibility (or high clinical suspicion) as PI-RADS scores 2–5, five as PI-RADS scores 3–5, three as PI-RADS scores 4–5, and two as PI-RADS score 5. Regarding magnet strength, 3 Tesla systems were most common, used in 21/32 studies, with 5/32 using 1.5 Tesla systems (two studies used both magnet strengths). Four did not report the magnet strength, and 24 did not report echo times. The majority of studies (26/32) assessed mRNA to derive transcriptomic data in relation to mpMRI signal and used most commonly microarray or RNAseq methods (18 studies). Protein-based studies were the second most common approach (8/32) with all studies using IHC, followed by studies using DNA sequencing (seven studies). Two studies looked at DNA methylation. In studies using mRNA, 22 used samples processed with formalin-fixed paraffin embedding, three used fresh frozen tissue, and six used fluid biomarkers. One study did not state the preparation method. Seven studies used macrodissection prior to genomic analysis, two used microdissection, and eight used neither (often, tissue punches), and in 15 studies, this was not applicable given the study methodology.

**Table 1:** Descriptive characteristics of included studies

Author	Year	Ref.	Cohort	*n*	Matched cohort	mpMRI scoring system	Visible definition	Invisible definition	Intermed. definition	DNA	DNA methylation	RNA	Protein	Genes	Platform	Preparation	Dissection	Tesla	Echo time (ms)
Lenkinski	2008	[46]	Radical	2	No	Suspicious/nonsusp	–	–	–	No	No	Yes	Yes	Multiple	Microarray/IHC	Fresh/frozen	Macro	3.0	165
Leyten	2013	[7]	Biopsy	115	No	Suspicious/nonsusp	–	–	–	No	No	Yes	No	*PCA3*	Commercial test	FFPE	–	3.0	–
Busetto	2013	[10]	Biopsy	171	No	Suspicious/nonsusp	–	–	–	No	No	Yes	No	*PCA3*	PCA3 assay	–	–	3.0	–
Renard-Penna	2015	[24]	Radical	106	No	PI-RADSv1	–	–	–	No	No	Yes	No	*CCP*	RT-PCR	FFPE	Macro	3.0	7–12
Kaufmann	2016	[9]	Biopsy	49	No	PI-RADSv1 sum score	≥7	<7	–	No	No	Yes	No	*PCA3*	PCA3 assay	–		1.5	–
Stoyanova	2016	[29]	Biopsy	6	No	Radiomic features	–	–	–	No	No	Yes	No	Multiple	Microarray	FFPE	Macro	3.0	2.8–83
McCann	2016	[44]	Radical	30	No	Radiomic features	–	–	–	No	No	No	Yes	*PTEN*	IHC	FFPE	–	3.0	–
De Luca	2016	[8]	Biopsy	282	No	PI-RADSv1	–	–	3	No	No	Yes	No	*PCA3*	PCA3 assay		–	1.5	–
Dulaney	2017	[27]	Biopsy	11	No	PI-RADSv2	5	1–4	–	No	No	Yes	No	Multiple	Microarray	FFPE	Micro	–	–
Lee	2017	[45]	Radical	48	No	PI-RADSv2	2–5	≤1	–	Yes	No	No	Yes	Multiple	FISH/sanger/IHC	FFPE	–	1.5/3.0	1.3–105.6
Leapman	2017	[13]	Biopsy	100	No	PI-RADSv1 (modified)	4–5	1–2	3	No	No	Yes	No	Oncotype	RT-PCR	FFPE	None	3.0	–
Jamshidi	2017	[35]	Radical	6	No	Suspicious/nonsusp	–	–	–	Yes	No	No	No	Multiple	Whole exome	FFPE	Macro	3.0	1.4–120
Palapattu	2017	[47]	Biopsy	31	No	Suspicious/nonsusp	–	–	–	Yes	No	Yes	Yes	Multiple	DNA/RNAseq/IHC	FFPE	–	3.0	–
Fenstermaker	2017	[11]	Biopsy	187	Yes	mSS	–	–	–	No	No	Yes	No	*PCA3*	PCA3 assay	–	–	3.0	–
Gronberg	2018	[12]	Biopsy	532	No	PI-RADSv2	3–5	2–1	–	No	No	No	Yes	*STHLM3*	Protein assay	–	–	1.5	–
Radtke	2018	[20]	Combo	11	No	PI-RADSv2	4–5	1–2	–	No	No	Yes	No	Multiple	Microarray	FFPE	Macro	3.0	–
Li	2018	[26]	Radical	16	No	PI-RADSv2	4–5	≤1	–	No	No	Yes	Yes	Multiple	RNAseq	FFPE	Macro	3.0	11–125
Kesch	2018	[32]	Biopsy	5	No	PI-RADSv1	–	–	–	Yes	Yes	No	No	Multiple	Methylation array	Not stated	–	3.0	–
Salmasi	2018	[14]	Combo	134	No	PI-RADSv2	–	–	–	No	No	Yes	No	Oncotype	Microarray	FFPE	–	3.0	–
Beksac	2018	[17]	Radical	206	No	PI-RADSv1	–	–	–	No	No	Yes	No	Multiple	Microarray	FFPE	None	3.0	–
Houlahan	2019	[2]	Radical	40	Yes	PI-RADSv2	5	1–2	–	Yes	No	Yes	No	Multiple	CNA/SNParray/RNAseq	FFPE	Macro	–	–
Parry	2019	[21]	Radical	6	No	PI-RADSv2	3–5	1–2	–	Yes	Yes	Yes	No	Multiple	Multiple	Fresh/frozen	None	1.5	64–107
Baumgartner	2019	[37]	Biopsy	53	Yes	PI-RADSv2	3–5	1–2	–	No	No	No	Yes	*PTEN*/*ERG*	IHC	FFPE	–	–	–
Purysko	2019	[19]	Radical	72	No	PI-RADSv2	3–5	1–2	–	No	No	Yes	No	Decipher	Microarray	FFPE	Micro	3.0	–
Hectors	2019	[30]	Radical	64	No	PI-RADSv1	–	–	–	No	No	Yes	No	Multiple	Microarray	FFPE	None	3.0	–
Martin	2019	[18]	Biopsy	102	Yes	PI-RADSv2	–	–	–	No	No	Yes	No	Decipher	Microarray	FFPE	–	3.0	–
Wibmer	2019	[25]	Biopsy	118	No	PI-RADSv2	–	–	–	No	No	Yes	No	*CCP*	Microarray	FFPE	–	3.0	7–120
Kornberg	2019	[15]	Biopsy	131	No	PI-RADSv2	–	–	–	No	No	Yes	No	Oncotype	Microarray	FFPE	–	3.0	–
Falagario	2019	[22]	Radical	520	No	Suspicious/nonsusp	–	–	–	No	No	Yes	No	Decipher	Microarray	–	None	1.5/3.0	–
Switlyk	2019	[43]	Combo	43	No	ADC	–	–	–	No	No	Yes	No	*PTEN*	Bead array, RT-PCR	Fresh/frozen	None	1.5	–
Sun	2019	[34]	Radical	6	No	Radiomic features	–	–	–	No	No	Yes	Yes	Multiple	RNAseq	FFPE	None	3.0	–
Salami	2019	[36]	Radical	10	No	PI-RADSv2	3–5	1–2	–	Yes	No	Yes	No	Multiple	Multiple	FFPE	None	–	–

ADC = apparent diffusion coefficient; CCP = cell cycle progression (Prolaris) score; CNA = circulating nucleic acid; Intermed. = intermediate/indeterminate score; ERG = ETS-related gene; FFPE = formalin-fixed paraffin embedded; FISH = fluorescence in situ hybridisation; IHC = immunohistochemistry; mpMRI = multiparametric magnetic resonance imaging; *N* = number of patients; PCA3 = prostate cancer antigen 3; PI-RADS = Prostate Imaging Reporting and Data System; PTEN = phosphatase and tensin homologue; Ref. = reference; RNAseq = RNA sequencing; RT-PCR = real-time polymerase chain reaction; SNParray = single nucleotide polymorphism array; STHLM3 = Stockholm3 test; susp. = suspicious.

### 3.2 Thematic synthesis

#### 3.2.1 Association of mpMRI conspicuity and clinically validated genetic biomarker panels

Validated commercial assays for the detection of prostate cancer or assessment of aggressive disease were investigated in 16 studies. Additionally, several larger-scale investigations used panels derived from these assays as part of their analysis.

Progensa prostate cancer antigen 3 (PCA3) is a prognostic marker that measures the ratio of *PCA3* to *PSA (KLK3)* mRNA and was found to be significantly higher in patients with mpMRI-visible tumours [7], [8]. In contrast, another study found no correlation between PCA3 level and tumour conspicuity; however, this study had a relatively small sample size (*n* = 49) [9]. Two other studies supported the use of PCA3 in conjunction with mpMRI to improve diagnostic accuracy significantly; however, they did not compare mpMRI-visible and mpMRI-invisible cancers [10], [11], and this was also true of the STKHLM3 assay [12].

Oncotype Dx genomic prostate score (GPS) is another prognostic marker, based on an RNA expression assay of 17 genes that is associated with pathological stage, grade, disease recurrence, and prostate cancer–specific mortality. Leapman and colleagues [13] found a significant association between GPS and prostate cancer visibility on mpMRI[13]. This association persisted only for men with significant disease (defined as Gleason score ≥ 3 + 4 cancer) [13]. These findings were reiterated in other studies describing an association between GPS and mpMRI visibility of clinically significant prostate cancer [14], [15].

Decipher, a genomic classifier (GC), is a 22-gene prognostic signature associated with early metastasis of prostate cancer [16]. Overall, mpMRI-visible tumours appear to have increased Decipher scores compared with mpMRI-invisible tumours, in both biopsy cohorts and radical prostatectomy cohorts [17], [18], [19], [20]. In contrast, two recent studies found no major association of a GC-based gene signature and tumour conspicuity on mpMRI; however, this may be attributed to a small sample size (*n* = 6) [21] and a low- to intermediate-risk cohort, mirroring similar results to studies using Oncotype DX in this patient population [13], [15], [22]. Additionally, another study found that GC added significant value to mpMRI in predicting adverse pathology upon radical prostatectomy, but did not correlate GC with mpMRI features directly [18]. In terms of radiogenomics, GC score was found to be most highly correlated with grey-level co-occurrence matrix (GLCM) texture, a measure of regularity and local spatial variation of intensity or colour brightness in an image to determine its texture [13], [23]. Thus, GC-related genes tend to correlate with mpMRI features, but, as with other candidate genes, only correlative studies have been performed without controlling for additional pathological factors that exist between mpMRI-visible and mpMRI-invisible tumours.

Finally, Prolaris cell cycle progression (CCP) is a prognostic gene signature comprising CCP-associated genes wherein each 1-unit increase in CCP score represents doubling of the risk of prostate cancer–specific mortality. PI-RADS was weakly correlated with CCP (*ρ* = 0.26, *p* = 0.007), but was able to predict a CCP score of >0 with sensitivity and specificity of 80.0% and 40.9%, respectively [24]. However, a small number of tumours with high CCP were overlooked by mpMRI [24]. Conversely, Wibmer et al [25] compared the CCP gene signature between mpMRI-visible and mpMRI-invisible cancers and found no significant difference [25]. Significant differences in CCP scores were, however, observed between patients with and without extracapsular disease extension on mpMRI [25].

#### 3.2.2 Association of mpMRI conspicuity with biological pathways and functions

Transcriptomic analysis was used in 18 studies to identify key pathways differing between mpMRI-visible and mpMRI-invisible tumours. Several studies used gene set enrichment analysis or over-representation analysis to identify enriched processes, pathways, or functions.

Pathways that regulate cell cycle and growth appear to be related to mpMRI conspicuity. Li and colleagues [26] reported enriched processes of mitotic cell cycle, protein folding, cell cycle, mitotic cell cycle process, and cell division in mpMRI-visible cancers. Furthermore, Dulaney and colleagues [27] reported that tumours with a PI-RADS score of 5 had significantly more deregulation of pathways involved in apoptosis and cell cycle (in particular, TGFβ, STAT, and RAS pathways) compared with mpMRI-invisible tumours; however, this was unadjusted for multiple testing and this study scored relatively low using the modified Newcastle-Ottawa scale (3/8), indicating a potential a risk of bias. Finally, Beksac et al [17] reported that pathways associated with CCP (PI3K-AKT-mTOR and E2F) and castration resistance (WNT-b) were found to be more active in mpMRI-visible cancer (PI-RADSv2 score of 5) than in mpMRI-invisible cancer.

Another major hallmark of aggressive cancer is evasion of immune destruction, and this was highlighted across several articles [28]. Stoyanova et al [29] reported increased immune/inflammatory and cell-stress responses in mpMRI-visible tumours in both the peripheral zone and the transitional zone, as derived through radiomic feature analysis. Another radiogenomic study reported significant enrichment of genes involved in immune responses in mpMRI-visible tumours, as defined by ADC GLCM energy-derived features [30]. As further indicative evidence of the immunological component of mpMRI conspicuity, Houlahan et al [2] reported a 200-fold increase in *ANKRD30A* (NY-BR-1; a tumour-specific antigen that selectively activates CD8 + T cells) in mpMRI-visible cancers.

DNA damage repair pathway defects play an important role in prostate cancer carcinogenesis and progression, and mutations are present in around 19% of prostate tumours of Gleason grade ≥ 8 [31]; these also appear to play a role in tumour conspicuity on mpMRI. Dulaney et al [27] noted significantly higher deregulation of DNA repair–related genes in mpMRI-visible targeted tumours with higher dynamic contrast enhancement values, as also noted in other studies [26]. Another case study found lower ADC values in tumour regions with a greater number of copy-number alterations and higher mutational burden [32]. Houlahan et al [2] also quantified genomic instability using the percentage of the genome altered (PGA) via copy-number alterations, finding elevated PGA in visible tumours (*p* = 0.03) with increased average length of individual amplifications and deletions. Tumour hypoxia is believed to be a characteristic driving cancer instability [33] and has been shown to correlate with mpMRI-derived radiomic features [34]. Contrasting this, a different study found no significant difference in mutation load in cancer-associated genes between regions that were histopathologically benign and had low clinical suspicion on mpMRI, intermediate clinical suspicion on mpMRI, and high-grade cancer histopathologically; however, this study was limited by its small sample size (*n* = 6) [35].

Lastly, gene sets involved in cell structure (eg, actin filament-based process and cytoskeleton organisation) were downregulated in mpMRI-invisible tumours, which may explain the physical properties (such as lower tissue density) associated with mpMRI-invisible cancer [26]. Salami et al [36] also identified an MRI-visibility signature comprising predominantly cell organisation/structure genes from 10 patients, which was able to distinguish MRI-visible tumours in an independent cohort with an area under the curve (AUC) of 0.88. This is further supported by the association of stromal-associated genes in the Oncotype DX assay being significantly associated with PI-RADS score, with little association seen in other gene groups [13].

#### 3.2.3 Association of mpMRI conspicuity with genetic markers for cancer aggressivity and prognosis

The association of *PTEN* loss (a known driver of prostate cancer) and mpMRI conspicuity was assessed in three included studies. *PTEN* loss was shown to be higher in mpMRI-targeted biopsies (ie, of mpMRI-visible tumours) than in non–image-guided systematic biopsies (ie, not of mpMRI-visible tumours) [37]. This result is concordant with the fact that *PTEN* loss is highly correlated with Gleason grade and stage [38], [39], [40] and that mpMRI-targeted biopsies detect more clinically significant tumours compared with systematic biopsies [1], [41], [42]. However, even when Gleason grade was controlled for, *PTEN* loss remained higher in the targeted biopsy group. A similar association between *PTEN* loss and ADC values was demonstrated in a radical prostatectomy population; however, no correlation between *PTEN* expression and Gleason grade was shown [43]. Other studies found an association with Gleason score (*r* = − 0.30, *p* = 0.04) and K^ep^ (*r* = − 0.35, *p* = 0.02) but not with ADC [44]. In contrast, a separate radical prostatectomy study found no association between *PTEN* and mpMRI characteristics; however, this study included PI-RADS score 2 tumours as visible, which may skew the study findings [45].

Li et al [26] performed a full-scale transcriptomic analysis of mpMRI-visible tumours compared with mpMRI-invisible tumours [26]. They found 1654 differentially expressed genes between these two visibility phenotypes. Expression of *CENPF*, *AGR2*, and *GDF15* was found to be enriched in mpMRI-visible tumours and was associated with reduced time to biochemical recurrence in an independent dataset, suggesting a potential link between mpMRI visibility and prognostic outcome [26]. *CENPF* (part of the Prolaris panel) was also suppressed using an inducible miRNA system in vivo, showing a reduction in mpMRI visibility when expression was reduced, suggesting a possible causal relationship between an identified gene and mpMRI conspicuity of prostate cancer [26]. Transcriptomic analysis also identified genes associated with tumour aggression in mpMRI-visible tumours, such as noncoding RNA *SCHLAP1* (linked to prostate cancer progression), several small nuclear RNAs [2], and angiogenesis factor *VEGF* [46]. Indeed, mutations in tumorigenic drivers such as *SPOP* and *IDH1* have been found even in lower-grade mpMRI-visible tumours [47].

One study derived an mRNA signature that could accurately predict visibility in both a training and a validation cohort (AUC = 0.89 and 0.88, respectively) but, when applied to The Cancer Genome Atlas cohort, found no significant differences in biochemical recurrence, distant metastasis, or cancer-specific mortality. However, this signature was derived and tested on a total of 26 patients, and the mpMRI visibility groups that were predicted did not significantly differ by Gleason grade, positive lymph nodes, or positive surgical margins, which somewhat contradicts other histopathological evidence [36], [48].

### 3.3 Bioinformatic synthesis

**Fig. 2:**
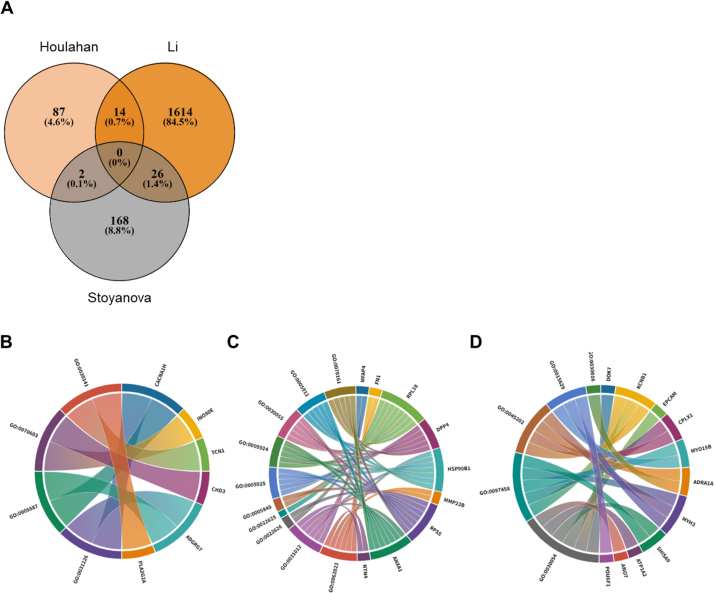
Bioinformatic synthesis of included studies. (A) Venn diagram of genetic overlap for mpMRI conspicuity–associated genes and (B–D) chord diagrams for each study (studies by Houlahan et al [2], Li et al [26], and Stoyanova et al [29], respectively) illustrating over-representation analysis of significant differentially expressed genes identified in each study and over-represented cellular component gene ontology terms associated with these genes. mpMRI = multiparametric magnetic resonance imaging.

We identified four studies with available data for bioinformatic analysis, three of which were large enough to compare the performance of gene panels [2], [26], [29]. All three studies used macrodissection of tumour tissue prior to nucleic acid extraction. For each study, we included a nonoverlapping list of significantly altered or significantly correlated genes. For example, in one study [29], a selection of genes were correlated with multiple radiological features; in this case, every gene that was significantly correlated with at least one radiological feature was included in our analysis (196 total). We identified 42 genes that demonstrated differential expression between mpMRI-visible and mpMRI-invisible tumours (in two or more of the included studies; Fig. 2A). Of note were *GDF15* and *AGR2*, which are purportedly involved in tumour progression [49], [50], [51], [52]. Interestingly, 14 of the identified MRI conspicuity–related genes were reported in studies that used a matched cohort methodology, suggesting that the influence of these genes may be independent of Gleason grade. Shared cellular components were over-represented in two studies [26], [29], namely, anchoring junction (*p* < 1.00E−15 and *p* = 0.0051), adherens junction (*p* = 1.34E−12 and *p* = 0.0041), focal adhesion (*p* = 1.34E−12 and *p* = 0.0041), cell-substrate adherens junction (*p* = 1.57E−12 and *p* = 0.0041), and cell-substrate junction (*p* = 2.11E-12 and *p* = 0.0041; Fig. 2). These cellular components are all involved in anchoring of cells to the extracellular matrix (ECM) or other cells, primarily through actin filaments or other components of the cytoskeleton. We found no significant over-representation of any components identified in one study [2] after FDR correction; the closest enriched component was actin-based cell projection (raw *p* = 0.0027, after FDR *p* = 0.67), further implicating cell-ECM interaction as a determinant of conspicuity.

From the derived themes, several panels of genes were suggested to be altered between mpMRI-visible and mpMRI-invisible tumours. In order to compare how matching for size and grade may alter this result, we assessed the Log2-fold change of each gene within the panels and RNAseq data from two studies: one that matched for Gleason grade and tumour volume [2], and one that did not match for these factors [26]. Overall, gene signatures had more significantly altered genes between mpMRI-visible and mpMRI-invisible tumours in the unmatched study [26] compared with the matched study [2], which suggests that their discriminant ability may derive from the association with Gleason grade and tumour size, rather than purely conspicuity. The effect sizes seen in unmatched study [26] also tended to be of greater magnitude.

### 3.4 Risk of bias

Overall, all included studies scored highly in the Newcastle-Ottawa scale, indicating an acceptably low risk of bias, with 24 studies scoring above five stars out of eight (Supplementary Fig. 1). All studies scored highly on patient selection; however, a potential bias was the prevalence of studies based on radical prostatectomy specimens, which reduced generalisability of findings. The second major risk of bias identified was the use of smaller-scale genetic investigations, with 17 studies using either small targeted panels or single gene investigations. Some of the included studies scored low (or zero) on outcome due to single-gene investigation methodology.

### 3.5 Discussion

Here, we have provided a large contemporary systematic review and bioinformatic analysis of the molecular evidence of prostate cancer conspicuity on mpMRI (Table 2). Visible mpMRI tumours are generally associated with genomic markers of disease aggressivity, including increased Decipher and Oncotype scores, and greater frequency of *PTEN* loss. This association is strengthened by increased enrichment of pro-proliferative signalling pathways, increased genome instability, DNA damage repair defects, and hypoxia in mpMRI-visible tumours. On balance, we found no overall, or comparable, genetic evidence of increased aggression in mpMRI-invisible tumours compared with that in mpMRI-visible tumours; however, there were infrequent, isolated reports of mpMRI-invisible prostate cancer bearing genomic hallmarks of aggressivity, which warrants future investigation.

**Table 2:** Summary of genetic features associated with tumour visibility on mpMRI

Feature type	Feature	Reference
Commercial assays	Progensa PCA3	[7], [8]
	Oncotype Dx	[13], [14], [15]
	Decipher (GC)	[17], [18], [19], [20]
	Prolaris (CCP)	[24]
DNA-related features	DNA repair defects	[26], [27]
	Copy-number alteration	[32]
	Mutational burden	[32]
	Genomic instability (PGA)	[2]
	*PTEN* loss	[37], [43], [44]
Transcriptomic features	Biochemical recurrence–associated genes (*CENPF*, *AGR2*, *GDF15*)	[26]
	Cancer progression–associated genes (*SCHLAP1*)	[2]
	Small nuclear RNAs	[2]
	Angiogenesis factor (*VEGF*)	[46]
	Tumorigenic drivers (*SPOP*, *IDH1*)	[47]
Biological hallmarks of cancer	Castration resistance (WNT)	[27]
	Immunological response	[2], [28], [29]
	Tumour hypoxia	[34]
	Tumour progression (*GDF15*, *AGR2*)	[2], [26], [29]
Biological pathways	Mitotic cell cycle	[26]
	Protein folding	[26]
	Cell cycle	[26], [27]
	Mitotic cell cycle process	[26]
	Cell division	[26]
	Apoptosis	[27]
	Cell cycle progression (PI3K-AKT-mTOR and E2F)	[27]
Cellular structure components	Actin filament-based process	[26]
	Cytoskeleton organisation	[26], [36]
	Stromal components	[13]
	Anchoring junction	[26], [29]
	Adherens junction	[26], [29]
	Focal adhesion	[26], [29]
	Cell-substrate adherens junction	[26], [29]
	Cell-substrate junction	[26], [29]
	Actin-based cell projection	[26], [29]

AGR2 = anterior gradient 2, a protein disulphide isomerase family member; AKT = AKT serine/threonine kinase; CENPF = centromere protein F; CCP = cell-cycle progression; E2F = E2F transcription factor; GC = genomic classifier; GDF15 = growth differentiation factor 15; IDH1 = isocitrate dehydrogenase (NADP + ) 1; mpMRI = multiparametric magnetic resonance imaging; mTOR = mechanistic target of rapamycin kinase; PCA3 = prostate cancer antigen 3; PGA = percentage of genome altered; PI3K = phosphoinositide 3-kinase, PTEN = phosphatase and tensin homologue; SCHLAP1 = SWI/SNF complex antagonist associated with prostate cancer 1; SPOP = speckle type BTB/POZ protein; VEGF = vascular endothelial growth factor; WNT = Wnt signalling pathway.

Transcriptomic data suggest that there is likely no single underlying biological process or pathway driving mpMRI visibility. However, cell-cell– and cell-ECM–associated genes exhibit differential expression between mpMRI-visible and mpMRI-invisible tumours, suggesting a possible explanation for the histopathological characteristics of prostate cancer conspicuity on mpMRI (including, cellular density).

Future research effort should focus on exploring the molecular basis of tumour visibility in larger patient cohorts. Indeed, the Re-IMAGINE trial (NCT04063566) will investigate the role of genetic biomarkers in conjunction with mpMRI for the diagnosis of prostate cancer and will provide important answers in this field. Furthermore, the current literature is skewed towards transcriptomic analysis, and may benefit from further DNA and epigenetic investigation.

Lastly, the studies included in this review used numeric radiological scoring systems (predominantly, Likert and PI-RADS) to define “visibility” and “invisibility”, and then compared genetic features between these two groups. As discussed, this methodology is fruitful to inform which features have higher enrichment in mpMRI-visible tumours than in mpMRI-invisible tumours. However, this approach does not necessarily provide a detailed description of the unique genetic features of what mpMRI-invisible disease may harbour, and dedicated research focussed primarily on disease invisibility is still warranted in the future. We also noted that many studies did not include detailed methodology around mpMRI scan acquisition, which could potentially affect the results; therefore, future studies may benefit from improved transparency to increase replicability.

It is increasingly apparent that tumour grade and size are not the only important histopathological determinants of tumour visibility and invisibility, with evidence that patterns such as intraductal carcinoma and cribriform pattern may have reduced visibility on mpMRI [53]. Unfortunately, a very small minority of the included studies in this review (4/32) used a matched cohort methodology, meaning that, in the majority of studies (28/32), the genetic influences on tumour conspicuity cannot be separated from the important influence that both tumour grade and volume have. Future studies would benefit from more rigorous histopathological matching [54] to help reveal the genetic aspects of disease conspicuity, beyond those associated with increased Gleason grade and tumour volume. However, this increases the difficulty in obtaining large sample numbers, particularly with continuous features such as tumour volume. Alternatively, following the advent of spatial transcriptomics, future research could use an internal matched control methodology, to potentially illuminate distinct genetic signatures in visible and invisible regions of the same prostate.

Lastly, mpMRI-visible tumours are more likely to have genetic variations that drive proliferation and therapeutic resistance. Therefore, if validated, mpMRI may have clinical utility in risk stratification and treatment selection, as tumour conspicuity may confer useful additional information, beyond tumour grade and size [4], [34]. Additionally, almost all current studies are correlative, and we found only a single instance whereby visibility-associated genes were verified in a model; as such, there is still extensive scope for future work to establish causative links [26].

## 4 Conclusions

Prostate cancer that is visible on mpMRI is generally enriched with molecular features of disease aggressivity and tumour development, including activation of proliferative signalling, DNA damage, and inflammatory processes. Bioinformatic analysis demonstrates concordant cellular components and biological processes associated with mpMRI conspicuity, which may in part account for the histopathological features of MRI-visible prostate cancer, such as higher Gleason grade disease and increased cellular density. Future radiobiological studies in this field should endeavour to use matched cohort-based methodology to elucidate genetic aspects of tumour conspicuity more clearly, when tumour size and grade are accounted for.

***Author contributions:*** Joseph M. Norris and Benjamin S. Simpson had full access to all the data in the study and takes responsibility for the integrity of the data and the accuracy of the data analysis.  

*Study concept and design:* Emberton, Norris, Parry, Simpson.

*Acquisition of data:* Kim, Norris, Simpson, You.

*Analysis and interpretation of data*: Norris, Simpson.

*Drafting of the manuscript*: Norris, Simpson.

*Critical revision of the manuscript for important intellectual content*: All authors.

*Statistical analysis:* Simpson.

*Obtaining funding:* Norris.

*Administrative, technical, or material support:* None.

*Supervision*: Emberton, Whitaker.

*Other:* None.  

***Financial disclosures:*** Joseph M. Norris certifies that all conflicts of interest, including specific financial interests and relationships and affiliations relevant to the subject matter or materials discussed in the manuscript (e.g. employment/affiliation, grants or funding, consultancies, honoraria, stock ownership or options, expert testimony, royalties, or patents filed, received, or pending), are the following: Norris receives funding from the Medical Research Council (MRC) and has previously received financial support for research from the National Institute of Health Research (NIHR), the Urology Foundation (TUF), and the Freemasons United Grand Lodge of England via the Royal College of Surgeons of England (RCSEng). Simpson receives funding from the Rosetrees Trust. Kirkham and Freeman have shares in Nuada Medical Ltd. Whitaker receives funding from Prostate Cancer UK, the Urology Foundation, and the Rosetrees Trust. Emberton receives funding from NIHR-i4i, MRC, Sonacare Inc., Trod Medical, Cancer Vaccine Institute, and Sophiris Biocorp for trials in prostate cancer; is a medical consultant to Sonacare Inc., Sophiris Biocorp, Steba Biotech, GSK, Exact Imaging, and Profound Medical; has stock interest in Nuada Medical Ltd; received travel allowance previously from Sanofi Aventis, Astellas, GSK, and Sonacare; and is a proctor for HIFU with Sonacare Inc. and is paid for training other surgeons in this procedure. The other authors declare no competing interests.  

***Funding/Support and role of the sponsor:*** Joseph M. Norris is funded by the 10.13039/501100000265MRC (Grant Reference: MR/S00680X/1). Benjamin S. Simpson is funded by the 10.13039/501100000833Rosetrees Trust.
