# Supplementary material for: Genetic Landscape of Prostate Cancer Conspicuity on Multiparametric Magnetic Resonance Imaging: A Systematic Review and Bioinformatic Analysis
Source: Eur Urol Open Sci. 2020 Aug 12;20:37–47. doi: 10.1016/j.euros.2020.06.006 (PMC7497895; doi:10.1016/j.euros.2020.06.006)
Supplement: Supplementary file 1 [file mmc1.docx]

**Supplementary Fig. 1 – Newcastle-Ottawa risk of bias assessment across included studies. Scores for selection (maximum of four points), comparability (maximum of two points) and outcome (maximum of two points) are shown across the 32 included studies. Outcome was adjusted to reflect the method used to compare genetic features. Criteria allowed a maximum of eight points.**


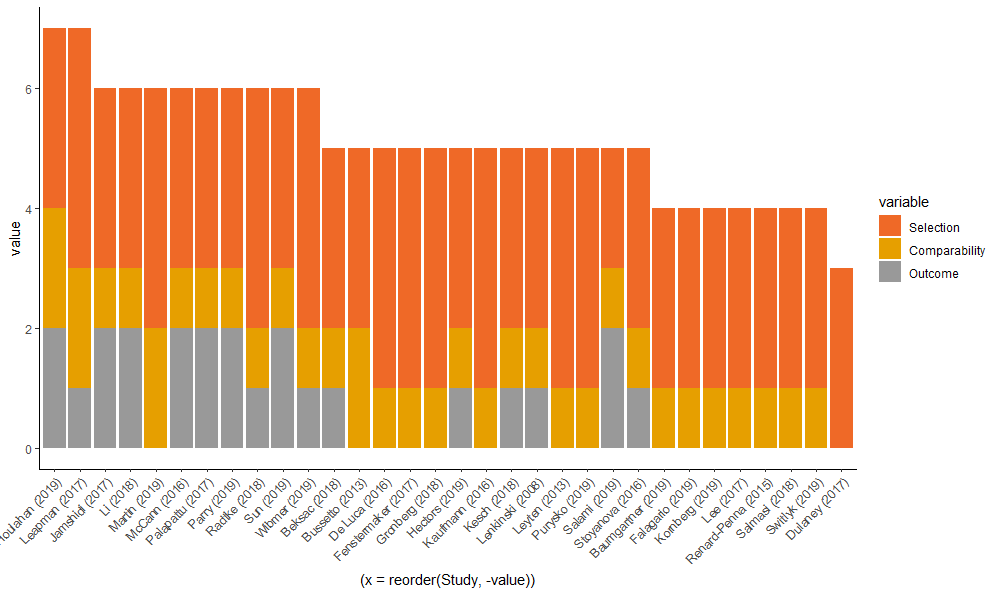


**Study**

**Score**

**Supplementary Fig. 2 – Over-representation analysis of 42 validated conspicuity-associated genes. Significant differentially expressed genes were analysed for over-representation in: A = biological functions; B = cellular components; C = molecular functions; D = KEGG pathways.Supplementary Fig. 3 – Over-representation analysis of conspicuity-associated genes as reported by Houlahan *et al.* Significant differentially expressed genes were analysed for over-representation in: A = biological functions; B = cellular components; C = molecular functions; D = KEGG pathways.**


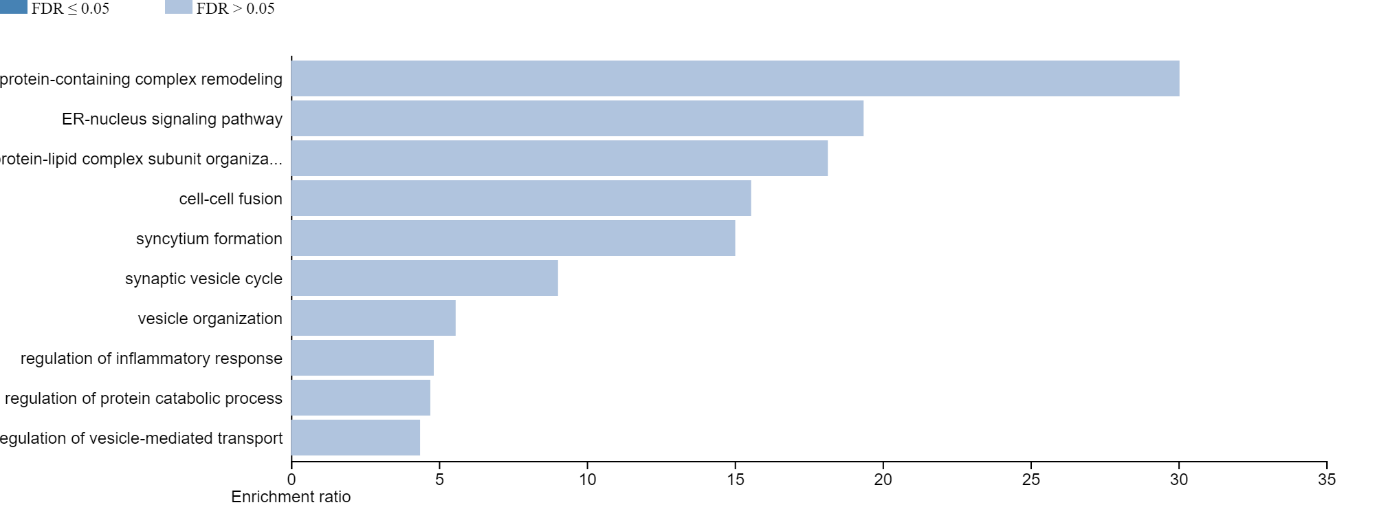

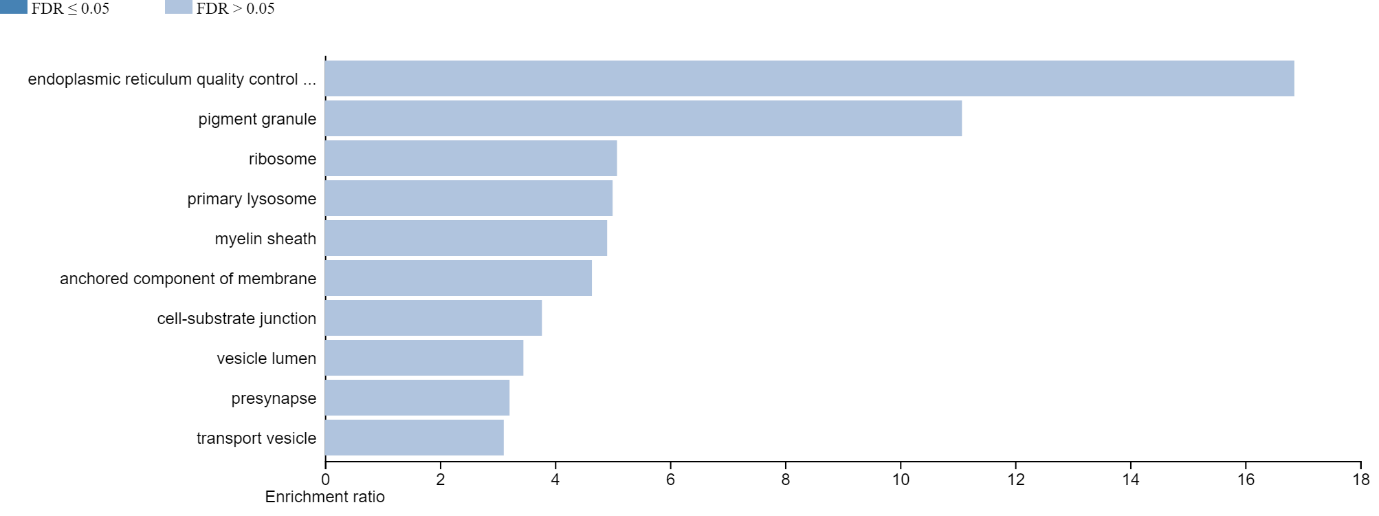

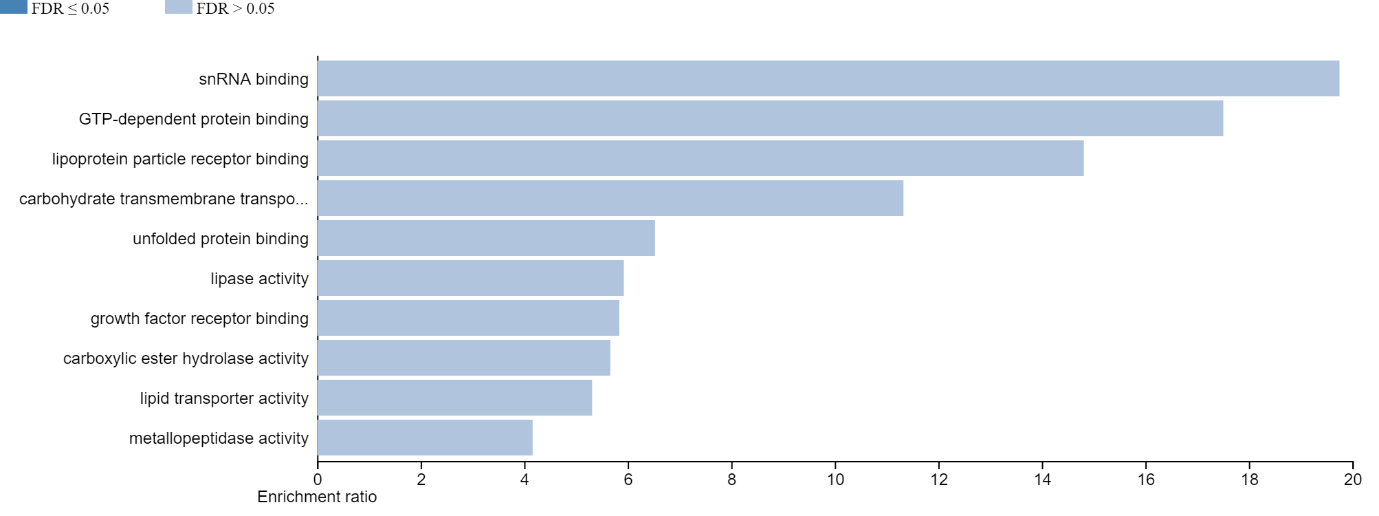

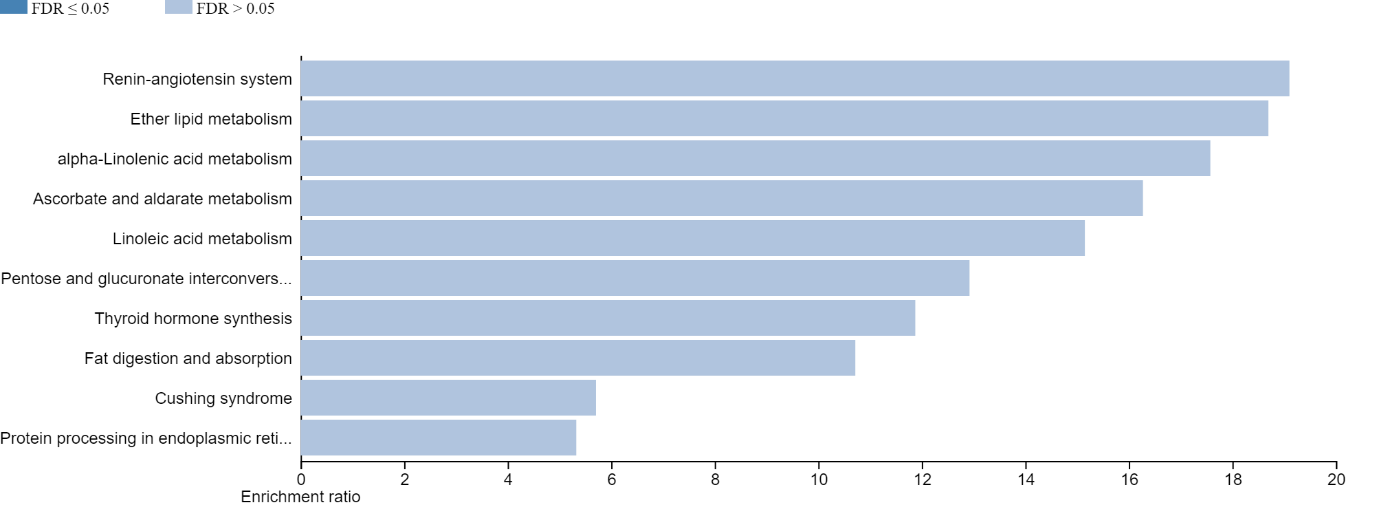


**A**

**B**

**C**

**D**


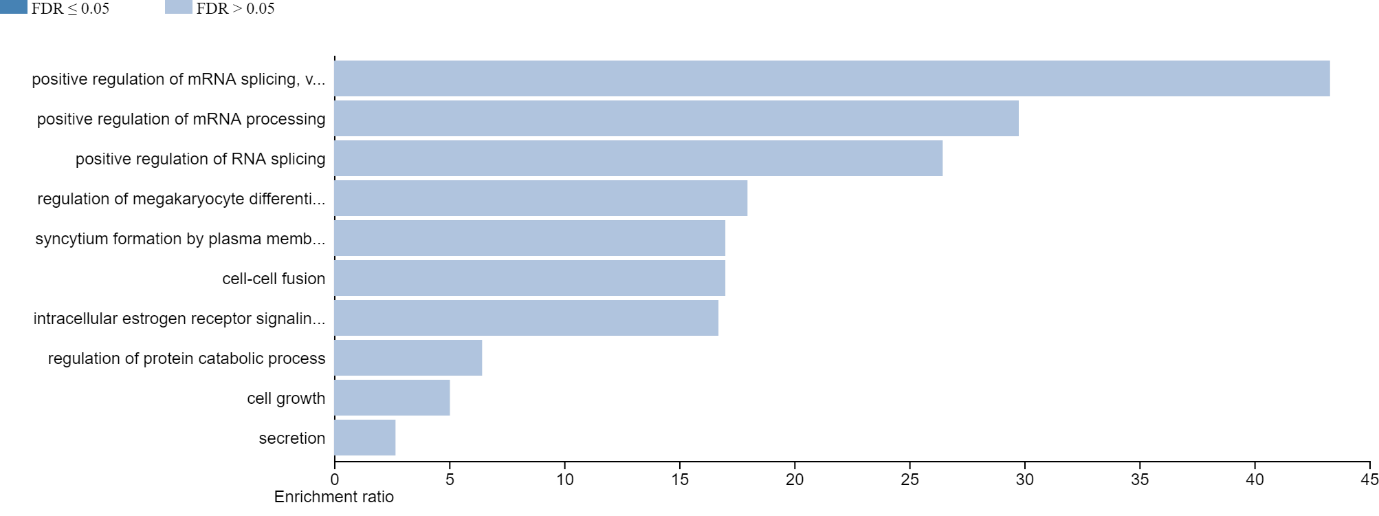

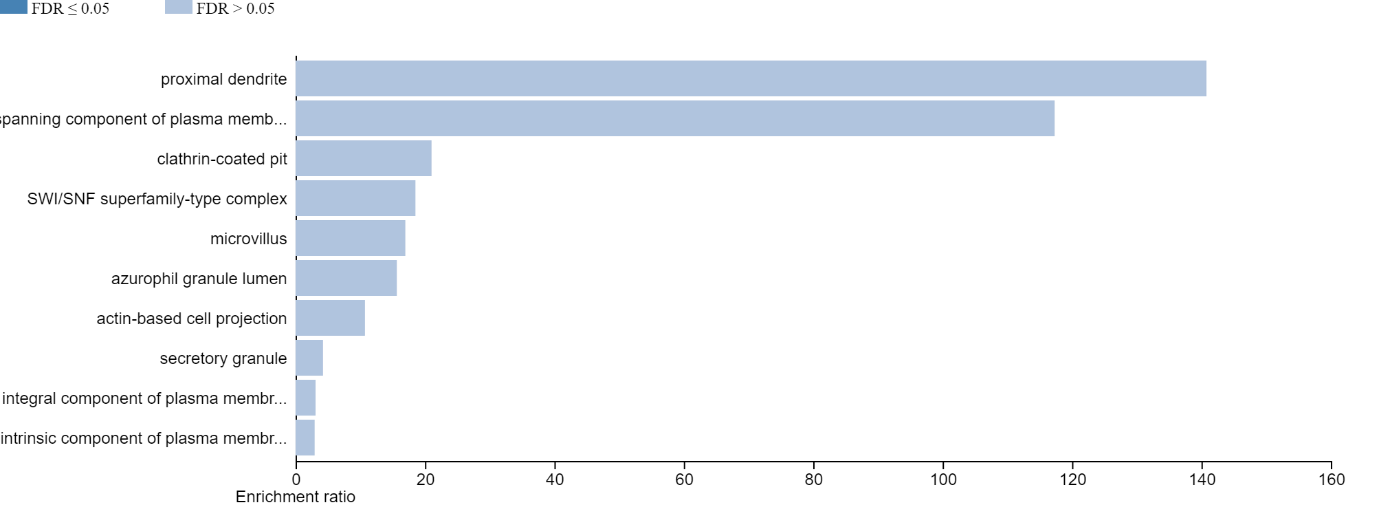

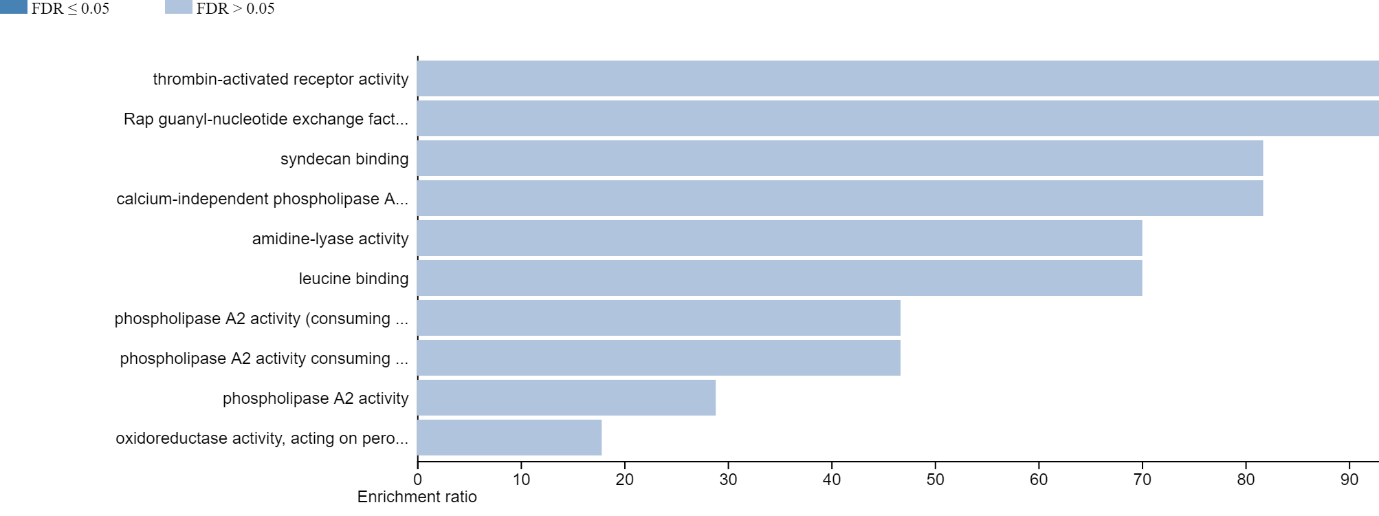

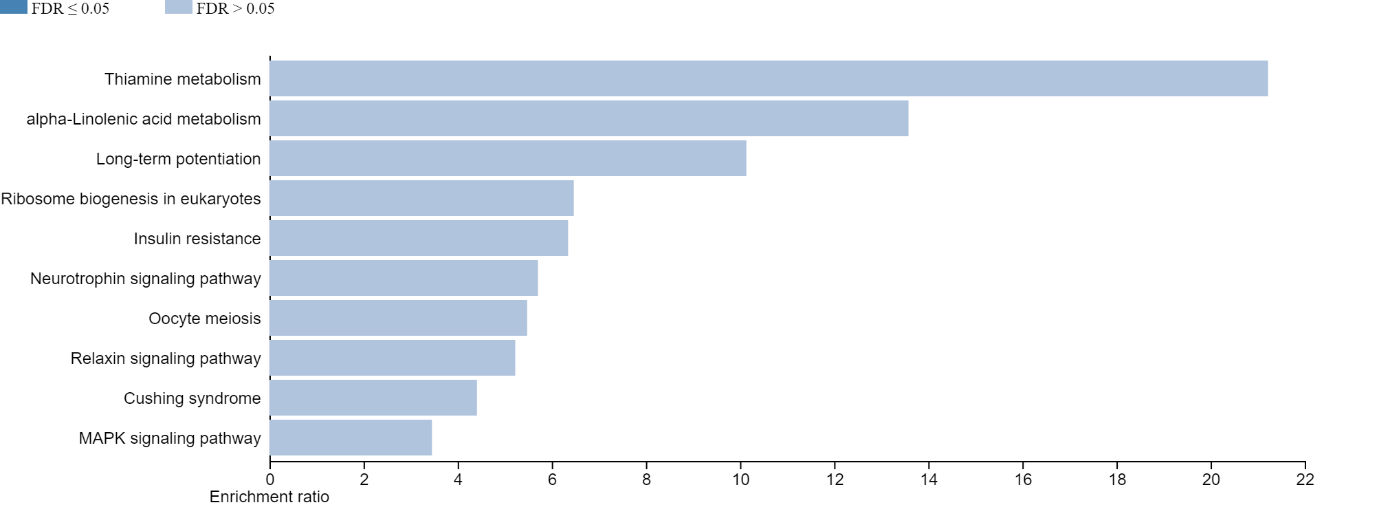


**A**

**B**

**C**

**D**

**Supplementary Fig. 4 – Over-representation analysis of conspicuity-associated genes as reported by Li *et al.* Significant differentially expressed genes were analysed for over-representation in: A = biological functions; B = cellular components; C = molecular functions; D = KEGG pathways.**


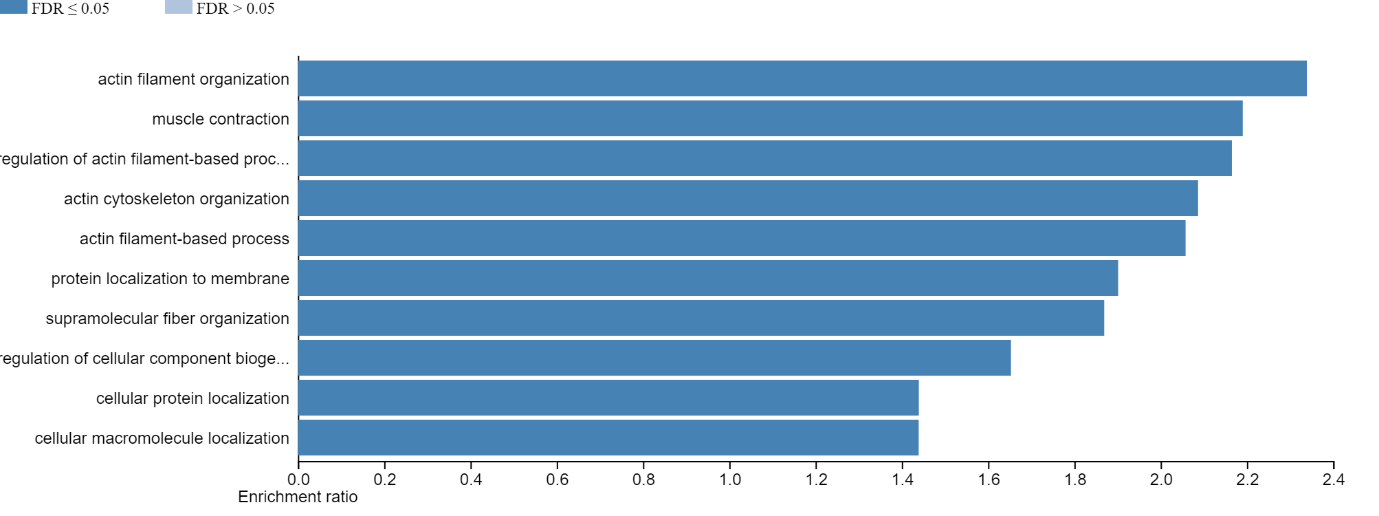

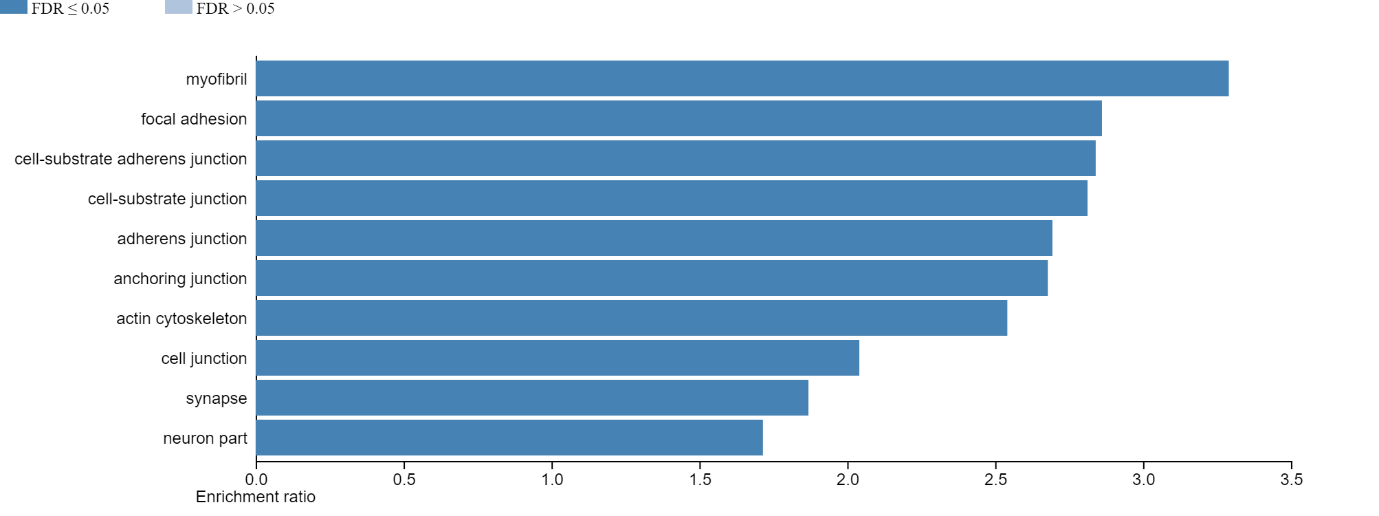

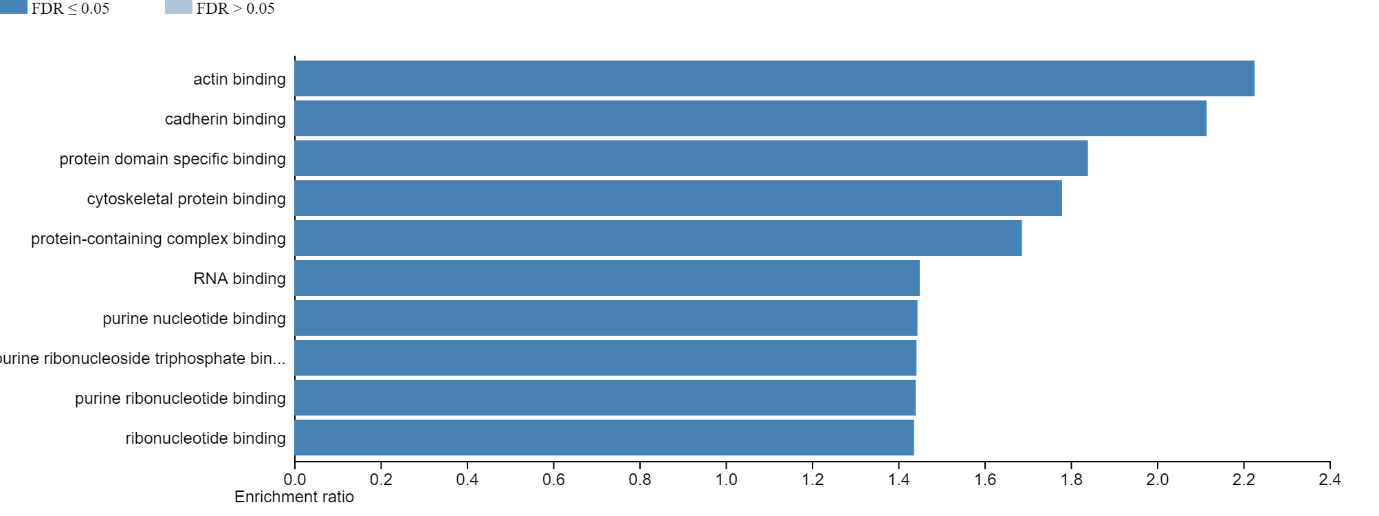

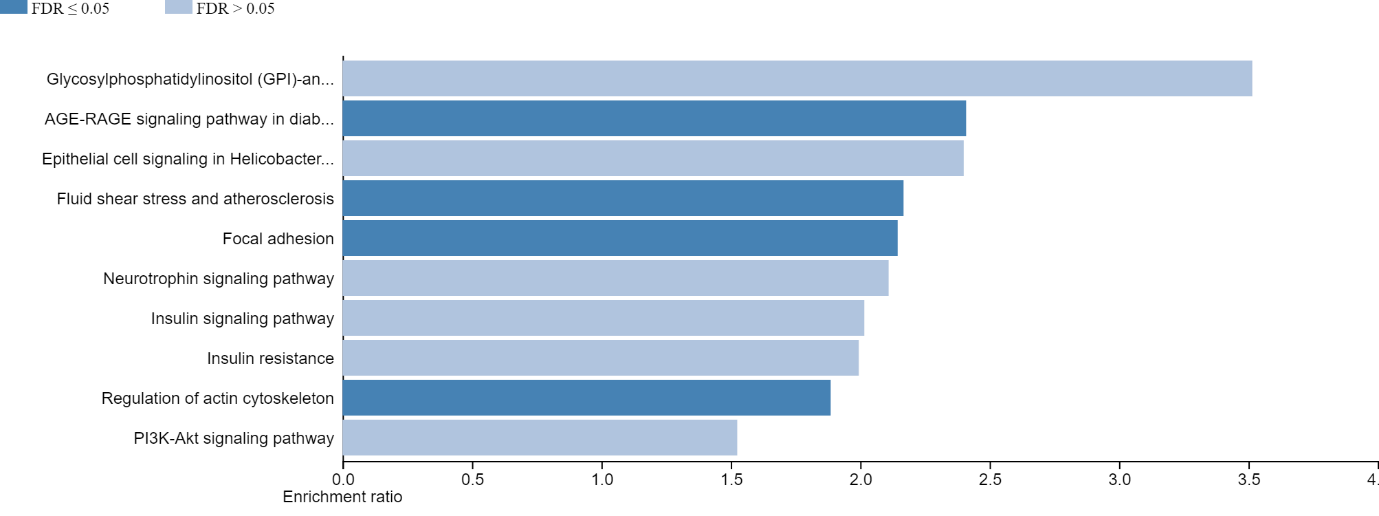


**A**

**B**

**C**

**D**

**Supplementary Fig. 5 – Over-representation analysis of conspicuity-associated genes as reported by Stoyanova *et al.* Significant differentially expressed genes were analysed for over-representation in: A = biological functions; B = cellular components; C = molecular functions; D = KEGG pathways.**


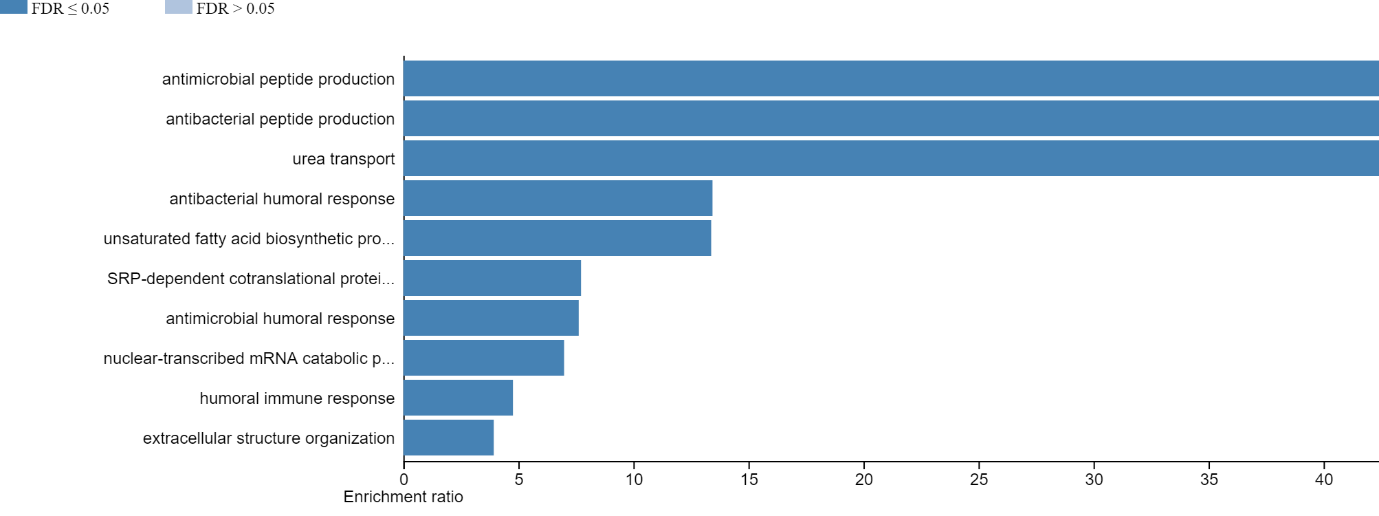

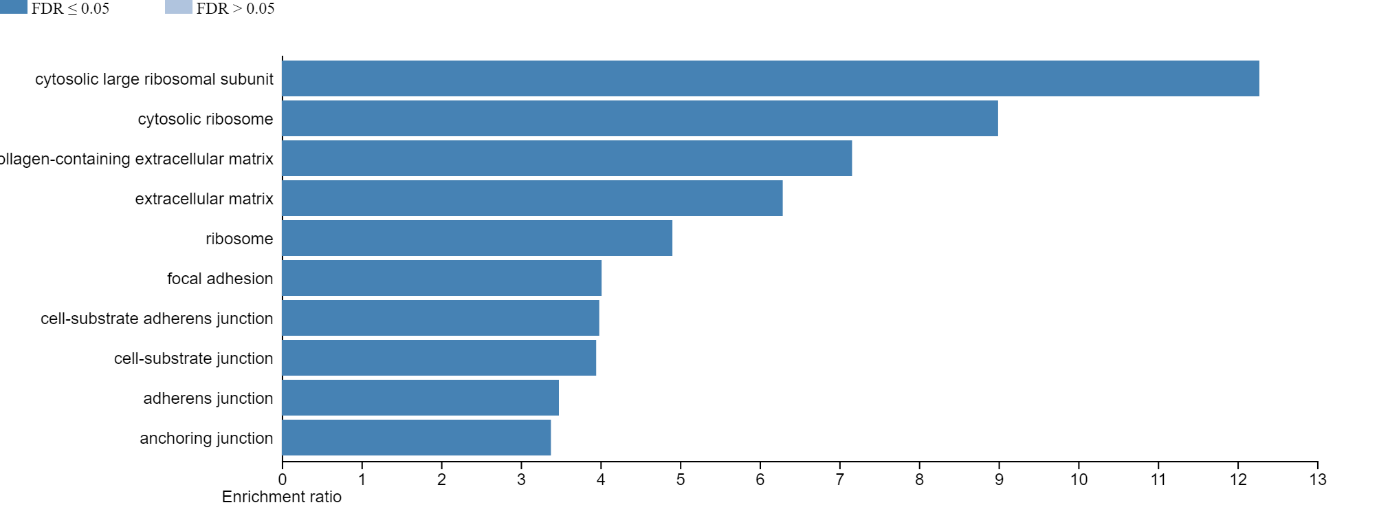

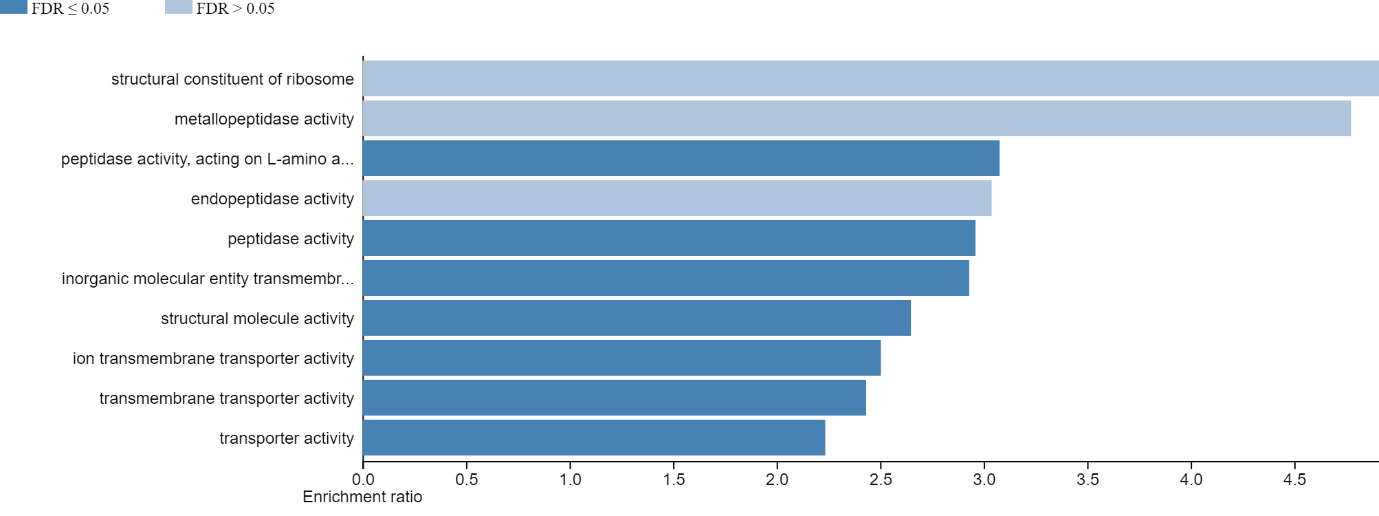

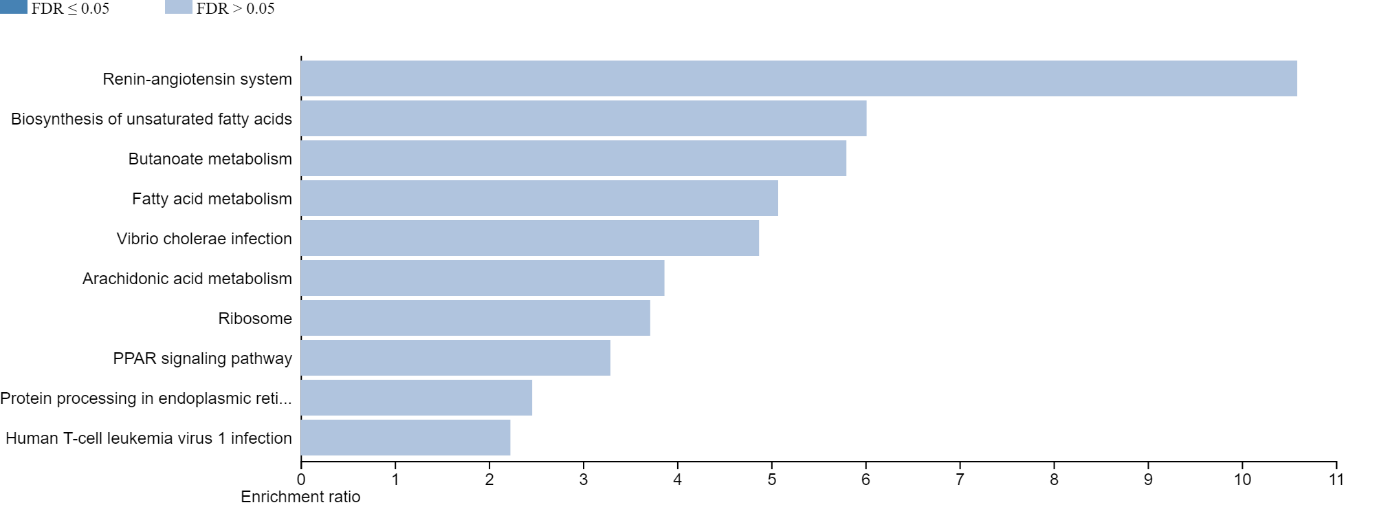


**A**

**B**

**C**

**D**
